# Linagliptin and secoisolariciresinol diglucoside attenuate hyperlipidemia and cardiac hypertrophy induced by a high-methionine diet in rats via suppression of hyperhomocysteinemia-induced endoplasmic reticulum stress

**DOI:** 10.3389/fphar.2023.1275730

**Published:** 2023-11-09

**Authors:** Israa A. Jalal, Abeer Elkhoely, Shimaa K. Mohamed, Amany A. E. Ahmed

**Affiliations:** Department of Pharmacology and Toxicology, Faculty of Pharmacy, Helwan University, Cairo, Egypt

**Keywords:** hyperhomocysteinemia, linagliptin, secoisolariciresinol diglucoside, GRP78, PERK, ATF-4, CHOP, SREPB1c

## Abstract

**Background:** Cardiac hypertrophy (CH) is one of the contributing causes of morbidity and mortality. Hyperhomocysteinemia (HHcy) is one of the diseases which may predispose hyperlipidemia and CH. Linagliptin (Lina) and secoisolariciresinol diglucoside (SDG) are known to alleviate a variety of illnesses by reducing oxidative stress and inflammation.

**Aim:** This study aimed to study the effect of HHcy on cardiac tissues, with a special focus on endoplasmic reticulum (ER) stress as a mainstay pathophysiological pathway. In addition, our study examined the protective effect of Lina, SDG, and their combination against HHcy-induced hyperlipidemia and CH in rats.

**Methods:** Seventy-five male Sprague–Dawley rats were randomly divided into five groups, and for 60 days, the following regimen was administered: Group I: rats received distilled water; Group II: rats received methionine (MET) (2 g/kg/day, p.o.); groups III and IV: rats received Lina (3 mg/kg/day, p.o.) and SDG (20 mg/kg/day, p.o.), respectively, followed by MET (2 g/kg/day, p.o.); Group V: rats received Lina and SDG, followed by MET (2 g/kg/day, p.o.).

**Results:** Pretreatment with Lina, SDG, and their combination showed a significant decrease in serum levels of HHcy and an improved lipid profile compared to the MET group. Moreover, both drugs improved cardiac injury, as evidenced by the substantial improvement in ECG parameters, morphological features of the cardiac muscle, and reduced serum levels of cardiac markers. Additionally, Lina and SDG significantly attenuated cardiac oxidative stress, inflammation, and apoptosis. Furthermore, Lina, SDG, and their combination remarkably downregulated the enhanced expression of endoplasmic reticulum (ER) stress markers, GRP78, PERK, ATF-4, CHOP, NF-κB, and SREBP1c compared to the MET-group.

**Conclusion:** Lina and SDG showed cardioprotective effects against HHcy-induced heart hypertrophy and hyperlipidemia in rats.

## 1 Introduction

Cardiovascular diseases (CVDs), such as heart failure (HF), are among the leading causes of death worldwide. Numerous risk factors may predispose to cardiovascular malfunctioning, including genetic make-up, gender, age, high blood pressure, hyperhomocysteinemia (HHcy), hyperlipidemia, hyperglycemia, and obesity (Liu et al., 2016; Francula-Zaninovic and Nola, 2018; Kim, 2021). A critical pathogenic mechanism in the development of HF is cardiac remodeling, in which cardiac hypertrophy (CH) is crucial (Qi et al., 2020).

Along with its well-known role in developing atherosclerosis, hyperlipidemia may directly impact heart dysfunction. Long-term hyperlipidemia leads to lipid accumulation in cardiac tissue, thus affecting the electrophysiological activity and heart function (Yao et al., 2020).

Among the medical conditions which may predispose hyperlipidemia is HHcy, a medical status specified as an abnormal blood concentration of the homocysteine (Hcy) amino acid. It may be caused by a deficiency of some enzymes incorporated in Hcy metabolism, especially cystathionine beta-synthase (CBS), methylenetetrahydrofolate reductase (MTHFR), and methionine sulfoxide reductase (MSR). Moreover, vitamin B deficiency, methionine (MET) overconsumption, and some drugs may also cause HHcy (Kim et al., 2018; Rehman et al., 2020).

MET, a necessary amino acid in animals, is metabolized into Hcy (Selhub et al., 1993). A high-MET diet is a well-established model for the development of HHcy and, consequently, the development of CVDs (Chwatko et al., 2007).

In the same context, HHcy-induced cardiac remodeling and HF may be a consequence of indirect mechanisms, specifically through predisposing dyslipidemia or a direct impact on cardiac tissues through activation of the myocardial redox state. HHcy starts the redox state either by an autoxidation reaction, producing reactive oxygen species, or indirectly by decreasing the expression of antioxidants (Majors et al., 1997; Racek et al., 2005; Mishra et al., 2009). One deleterious outcome predisposed by oxidative stress is the misfolding of proteins traversing the endoplasmic reticulum (ER), leading to ER stress, which occurs when the capacity of ER to fold protein is overwhelmed (Outinen et al., 1998).

ER stress is critically involved in the pathophysiology of many diseases (Ozcan and Tabas, 2012). Previous studies have shown that increased intracellular Hcy levels boost the ER stress response genes like glucose-regulated protein 78 (GRP78) (Werstuck et al., 2001; Colgan et al., 2007). In response to ER stress, ER transmembrane signal transducers, inositol-requiring enzyme 1 (IRE1), protein kinase RNA-like endoplasmic reticulum kinase (PERK), and activating transcription factor 6 (ATF6) are activated (Wang et al., 2009). The activation of PERK leads to the enhancement of eukaryotic translation-initiation factor 2α (eIF2α), followed by ATF4 upregulation, which, in turn, accelerates the expression of C/EBP homologous protein (CHOP), a proapoptotic transcription factor (Outinen et al., 1998). Moreover, activated ATF4 leads to ROS generation and NF-κB activation, resulting in oxidative stress and inflammation (Zhang and Kaufman, 2008).

Additionally, ER stress activates sterol regulatory element-binding proteins (SREBPs), transcription factors responsible for the upregulation of genes implicated in lipid production (Dorotea et al., 2020). In the liver, SREBP activation regulates the genes accountable for triglyceride biosynthesis and the expression of HMG-CoA reductase (3-hydroxy-3-methyl-glutaryl-coenzyme A reductase), the rate-limiting enzyme in cholesterol biosynthesis (Zhou and Austin, 2009). Regarding HDL, Hcy lowers HDL cholesterol plasma levels by blocking the hepatic synthesis of apo A-I, the primary HDL apolipoprotein (Barter and Rye, 2006).

Moreover, ER stress accelerates SREBP expression in the cardiac tissues, elevating intracellular lipid levels and cardiac lipidopathy (Colgan et al., 2007). Elevated intracellular fat levels increase lipolysis of cardiac lipids and oxidation of fatty acids, leading to cardiac mitochondrial dysregulation and exacerbated ER stress, thus forming a vicious loop of lipid biosynthesis, mitochondrial dysfunction, and ER stress, leading to ventricular malfunction (Ayyappan et al., 2020).

Linagliptin (Lina) is a dipeptidyl peptidase-4 (DPP4) inhibitor that maintains incretin hormones, such as gastric inhibitory polypeptide (GIP) and glucagon-like peptide GLP-1, by inhibiting their degradation mediated by dipeptidyl peptidase 4, thus reducing postprandial insulin secretion and inhibiting apoptosis of pancreatic cells (Karaca et al., 2009). Studies indicate that Lina has a wide range of effects in addition to its glycemic actions. Based on many studies, Lina significantly ameliorated dyslipidemia and cardiac fibrosis (Aykan et al., 2019; Cuijpers et al., 2021). Additionally, Lina improved antioxidant status (Koibuchi et al., 2014) and alleviated ER stress in hepatic and brain tissues by reducing expression levels of both GRP78 and CHOP messenger RNA (El-Deeb et al., 2020; Santos et al., 2020).

Secoisolariciresinol diglucoside (SDG), a lignan isolated from flaxseed, has satisfactory effects on CVDs, cancer, metabolic syndrome, and obesity (Rom et al., 2018). Regarding hyperlipidemia, SDG reduces oxidative stress, total blood cholesterol, and LDL-C levels, and raises serum HDL-C levels (Wei et al., 2020). Furthermore, SDG restores ER stress homeostasis by reducing the expression of ER stress mediators, IRE1α, Bip, CHOP, PERK, and ATF6 (Wei et al., 2020).

The current study aimed to explore the mechanisms underlying HHcy-induced hyperlipidemia and CH with a deeper insight into ER stress, which, to the best of our knowledge, is the first to be evaluated in the cardiac tissue in the case of HHcy. Moreover, our study is extended to investigate the effect of Lina, SDG, and their combination on HHcy-induced hyperlipidemia and cardiac dysfunction in the rat model in the absence of other cardiovascular risk factors.

## 2 Materials and method

### 2.1 Experimental animals and ethics

Seventy-five adult male Sprague–Dawley rats, weighing 180–200 g, were purchased from the breeding unit of the Egyptian Organization of Biological Products and Vaccines (Helwan, Egypt). Animals were acclimatized for 7 days under standard laboratory conditions at 25°C ± 2°C and 12 h light/dark cycling. They were fed an *ad libitum* standard pellet diet and water.

The research ethics and animal care and use committee of the Faculty of Pharmacy, Helwan University (protocol number: 02A2021), approved the experimental techniques and animal care.

### 2.2 Chemicals

Methionine powder (purity 98%) was obtained from Xian Plant Bio-Engineering Co., (Shaanxi, China) (catalog no. PLT-AA-17). It was suspended in 1% Tween 80 (Sigma-Aldrich Chemicals Co., St. Louis, MO, United States) in distilled water. Secoisolariciresinol diglucoside (SDG) powder (purity 60%) was obtained from Xian Plant Bio-Engineering Co., (Shaanxi, China) (catalog no. PLT-59). Linagliptin powder (purity 99.5%) was obtained from Nanjing Yanst Bio-Tech. Co., (Nanjing, China) (batch no. FI201118) dissolved in 1% Tween 80 in distilled water.

### 2.3 Groups and treatment

#### 2.3.1 HHcy induction

For induction of HHcy, rats were orally administered MET (2 g/kg/day, p.o.) suspended in 1% Tween 80 in distilled water for 60 days. The dose selection and the experimental duration were based on both a preliminary study and previous research (Bonaventura et al., 2009). HHcy was determined by measuring the plasma Hcy level.

#### 2.3.2 Experimental design

**FIGURE 1:**
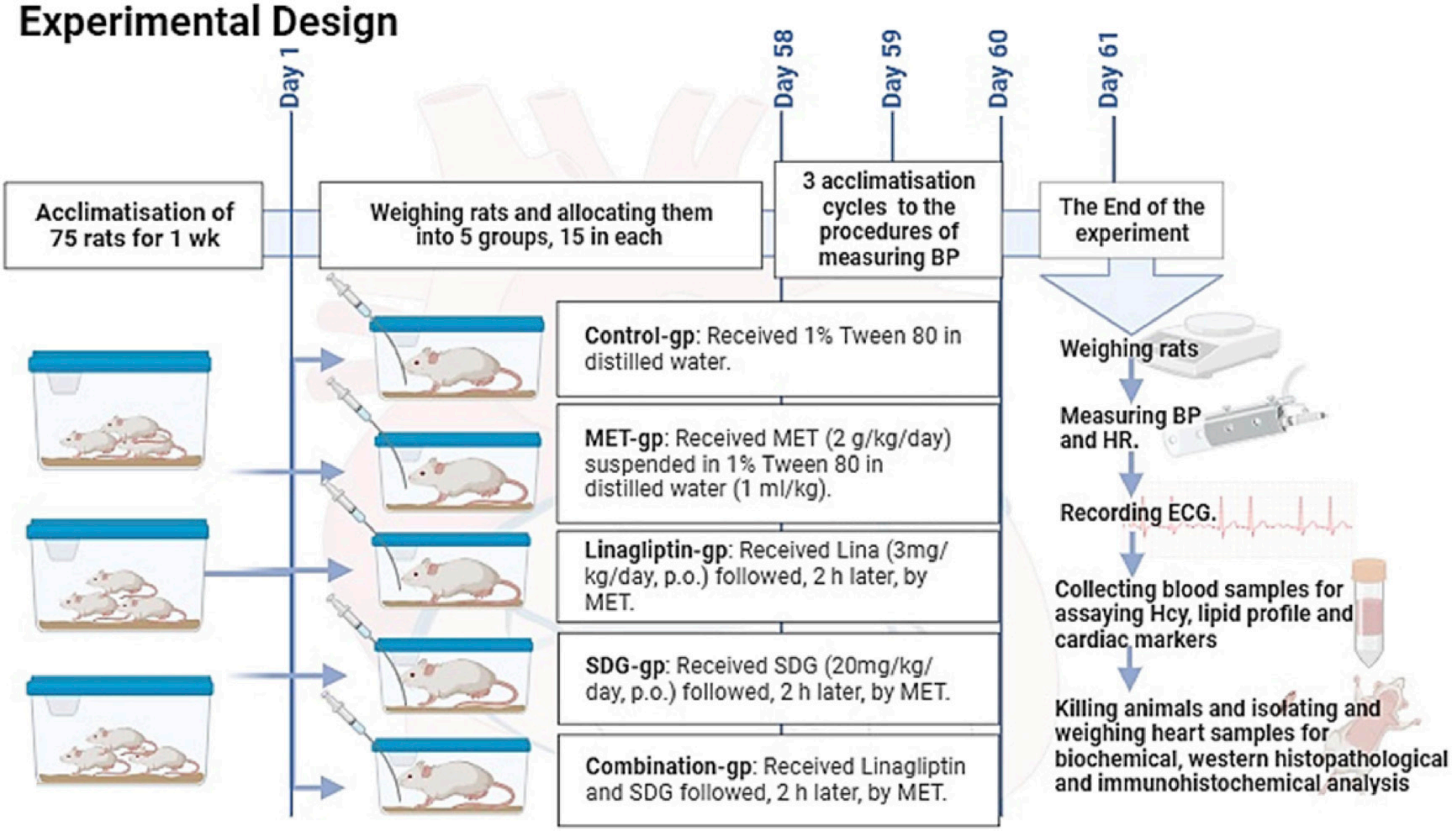
Experimental design of the study.

After acclimatization, 75 adult male Sprague–Dawley rats weighing 180–220 g, aged 6–8 weeks, were randomly allocated into five groups, with 15 rats in each group (Figure 1).

##### 2.3.2.1 Group 1: control group

It included 15 rats that received 1% Tween 80 in distilled water and were fed an *ad libitum* commercial rodent diet and water for 60 days.

##### 2.3.2.2 Group 2: MET-treated group

It included 15 rats that received MET (2 g/kg/day, p.o.) for 60 days.

##### 2.3.2.3 Group 3: Lina-treated group

It included 15 rats that received Lina (3 mg/kg/day, p.o.), followed, 2 h later, by MET (2 g/kg/day, p.o.) for 60 days. The dose selection was based on Si et al. (2019).

##### 2.3.2.4 Group 4: SDG-treated group

It included 15 rats that were administered SDG (20 mg/kg/day, p.o.), followed, 2 h later, by 2 g/kg/day MET (2 g/kg/day, p.o.) for 60 days. The dose selection was based on Zanwar et al. (2014).

##### 2.3.2.5 Group 5: Lina + SDG-treated group

It included 15 rats that received both Lina (3 mg/kg/day, p.o.) and SDG (20 mg/kg/day, p.o.), followed, 2 h later, by MET (2 g/kg/day, p.o.) for 60 days.

### 2.4 Hemodynamic measurements

The rats were weighed at the beginning of the experiment and after 60 days. After three acclimatization cycles, blood pressure (BP) and heart rate (HR) were measured 24 h after the last dose using a CODA multichannel, computerized, noninvasive BP device (Kent Scientific Co., Torrington, CT, United States).

Chlorpromazine (0.75 mg/kg, I.P.) (Sigma-Aldrich Chemicals Co., St. Louis, MO, United States) (product no. BP856) and ketamine (100 mg/kg, I.P.) (Sigma-Aldrich Chemicals Co., St. Louis, MO, United States) (product no. Y0000450) were then used to anesthetize the rats (Ambrosi et al., 2016). Then, the electrocardiogram (ECG) was recorded using a digital acquisition system PowerLab 4/30, laboratory chart software (ADInstruments Inc., Dunedin, New Zealand).

Afterward, blood samples were taken from the orbital sinus of the rats. Centrifugation at 4,000 rpm for 10 min was performed to separate the serum, which was then stored at –80°C to be used for the assessment of Hcy, lipid profile, and cardiac markers.

After obtaining blood samples, the animals were euthanized by cervical dislocation, and an abdominal incision was made along the midline. The hearts were then quickly removed, washed in ice-cold phosphate-buffered saline (El-Nasr Chemical Co., Abu Zaabal, Cairo, Egypt), dried, and weighed to determine the heart-to-body weight ratio (HW/BW). From each group, six hearts were homogenized in phosphate-buffered saline, and the supernatants were kept at–80°C. The homogenates were centrifuged at 5,000 rpm for 5 min at 4° C, following two freeze–thaw cycles to disrupt the cell membranes, and the clear supernatants were used for the biochemical examination. Another six hearts from each group were homogenized in RIPA lysis buffer, and the clear supernatants were used for Western blot analysis.

Finally, three heart samples from each group were fixed in 10% phosphate-buffered formalin for histopathological and immunohistochemical analyses.

### 2.5 Biochemical serum analysis

Hcy and cardiac troponin I (cTn-I) levels were measured using CUSABIO ELISA kits (catalog nos CSB-E13376r and CSB-E08594r, respectively) (Wuhan, China). The minimum detectable doses of rat Hcy and cTn-I are typically less than 0.195 nmol/mL and 7.81 pg/mL, respectively.

The total cholesterol (TC) level was measured using BioAssay Systems EnzyChrom^TM^ AF Cholesterol Assay Kit (catalog no. E2CH-100) (Hayward, CA, United States). The linear detection range for the colorimetric assay is 0.1–10 mg/dL cholesterol.

Triglyceride (TG) and HDL-C levels were measured using BioMed diagnostic commercial kits (OR, United States) (catalog nos TG117090 and CKM108025, respectively). CK-MB activity was measured using colorimetric BioDiagnostic commercial kits (catalog no. CH 12 30) (Giza, Egypt). The sensitivities of these methods are 3 mg/dL, 1 mg/dL, and 1 U/L for TG, HDL-C, and CK-MB, respectively. All the procedures were carried out according to the provider’s instructions.

Moreover, the atherogenic index and atherogenic coefficient were used to predict CVD risks. TG and HDL-C make up the unique index known as the atherogenic index of plasma (AIP). It has been used to measure blood lipid levels and is frequently considered the best indicator of dyslipidemia and related disorders (such as cardiovascular diseases). The atherogenic coefficient (AC) is a different index that is determined by the non-HDL-C to HDL-C ratio (Singh, 2015).

### 2.6 Biochemical assay of heart tissues

Reduced glutathione (GSH) and malondialdehyde (MDA) contents in the heart homogenate supernatant were determined using a colorimetric MyBioSource research kit (catalog no. MBS265966) and BioVision (Milpitas, CA, United States) (catalog no. K739-100), respectively. The sensitivities of these methods for GSH and MDA are up to 0.5 ug/mL and 0.1 nmol/well, respectively.

Inflammatory markers interleukin 8 (IL-8) and TNF-α contents were measured using MyBioSource rat enzyme-linked immunosorbent assay kits (ELISA) (C.A., United States) (catalog nos MBS9141543 and MBS2507393, respectively). The sensitivities of the assays for both IL-8 and TNF-α are approximately 1.0 pg/mL and 46.88 pg/mL, respectively. All the procedures were carried out according to the provider’s instructions. The Elabscience Rat ANP (atrial natriuretic peptide) ELISA kit (TX, United States) (catalog no. E-EL-R0017) was used to determine the quantity of ANP in the heart homogenate supernatant. The processes were in accordance with the vendor’s guidelines. The sensitivity of this assay is approximately 9.38 pg/mL.

### 2.7 Histopathological observation

Sections of cardiac tissues were fixed in 10% formal saline for 72 h. Dehydration was achieved by washing in tap water, followed by successive dilutions of alcohol. Samples were cleared in xylene before being embedded in paraffin for 24 h at 56° C in a hot air oven. Paraffin beeswax tissue blocks were made for sectioning at 50 µm thickness for hearts and stained with hematoxylin and eosin (H&E) (Culling, 1974). The slides were observed under a light microscope (at ×400 magnification).

### 2.8 Immunohistochemical analysis

Paraffin-embedded tissue sections having a thickness of 5 microns were obtained per the manufacturer’s protocol. According to Elsayed et al. (2022), sections of the deparaffinated recovered tissue were exposed to 0.3% H_2_O_2_ for 20 min. Afterward, anti-cleaved caspase-3 (GB11532, Servicebio–1: 500) was incubated with cardiac samples overnight at 4°C. Afterward, tissue sections were treated with the secondary antibody HRP EnVision kit (DAKO) for 20 min before being cleaned again and incubated with diaminobenzidine (DAB) for 15 min. After being cleaned with PBS, the samples were counter-stained with hematoxylin, dehydrated, and cleared with xylene before being covered for microscopic inspection.

In the immunohistochemically stained sections, the percentage area of expression levels of cleaved caspase-3 was determined in at least six randomly chosen, non-overlapping fields for each sample. Utilizing the Leica application module for histological analysis coupled to the full HD microscopic imaging system (Leica Microsystems GmbH, Germany), all light microscopic examinations were performed, and data were collected.

### 2.9 Western blot analysis

Proteins were extracted from heart samples and estimated using the RIPA lysis buffer and the Bradford protein assay kit (Bio Basic Inc.). Proteins were then electrophoresed on a sodium dodecyl sulfate–polyacrylamide gel and transferred to a polyvinylidene difluoride membrane with the Bio-Rad Trans-Blot Turbo apparatus. The membranes were incubated with primary antibodies against ATF4, CHOP, GRP78, PERK, and SREBP1c (Thermo Fisher Scientific, Waltham, MA, United States) (antibodies’ catalog nos: PA5-40294, PA5-104528, PA1-014A, PA5-40294, and PA1-014A, respectively). Afterward, membranes were washed with Tris-buffered saline with Tween 20 (TBST) buffer and then incubated with the goat anti-rabbit HRP-conjugated secondary antibody solution (Novus Biologicals, Littleton, CO, United States), and finally rinsed in TBST buffer. According to the manufacturer’s instructions, the chemiluminescent substrate (Clarity Western ECL substrate, Bio-Rad, Hercules, CA, United States) was applied to the blot. A CCD camera-based imager was used to visualize the protein bands. A Chemi Doc MP imager was used to analyze the images, and they were normalized using β-actin protein expression.

### 2.10 Statistical analysis

The findings were statistically analyzed using GraphPad Prism version 9.1 software (GraphPad Software, San Diego, CA, United States). The results were expressed as mean ± standard deviation (S.D.). One-way analysis of variance (ANOVA) was used to assess significance between the groups, followed by Tukey’s multiple comparison *post hoc* test. A *p*-value of less than 0.05 was considered significant.

## 3 Results

### 3.1 Effects of Lina, SDG, and their combination on the Hcy level, hemodynamic measurements, and the ratio of heart weight to body weight

Administration of MET showed a significant increase in the Hcy serum level by 3.6-fold compared to that in the control group (Table 1). Nevertheless, pretreatment with Lina, SDG, and combined Lina + SDG showed a significant decrease in the Hcy serum level by 41.3%, 41.1%, and 60.3%, respectively, compared to that in the MET group.

**TABLE 1:** Effects of Lina (3 mg/kg/day. p.o.), SDG (20 mg/kg/day, p.o.), and their combination on HW/BW, the Hcy level, blood pressure, and heart rate in MET-treated rats.

Parameter	Control	MET	Lina	SDG	Lina + SDG
Hcy level (nmol/mL)	3.535 ± 0.4388	12.85 ± 0.5828^a^	7.545 ± 0.3746^a^ ^,^ ^b^	7.573 ± 1.083^a^ ^,^ ^b^	5.102 ± 0.6539^a^ ^,^ ^b^ ^,^ ^c^ ^,^ ^d^
Body weight (g)	234.7 ± 16.46	245.8 ± 23.51	242.5 ± 9.24	241.0 ± 14.6	228.6 ± 12.19
Heart weight (mg)	624.9 ± 63.49	999.8 ± 18.06^a^	781.6 ± 52.51^a^ ^,^ ^b^	798.1 ± 45.40^a^ ^,^ ^b^	698.3 ± 40.18^b^ ^,^ ^c^ ^,^ ^d^
HW/BW (mg/g)	2.665 ± 0.1056	4.105 ± 0.3630^a^	3.235 ± 0.1261^a^ ^,^ ^b^	3.323 ± 0.1213^a^ ^,^ ^b^	3.043 ± 0.04383^a^ ^,^ ^b^
SBP (mmHg)	119.9 ± 4.166	130.5 ± 8.855	126.6 ± 8.025	128.7 ± 9.067	120.4 ± 2.667
DBP (mmHg)	89.8 ± 7.262	100.1 ± 7.304	96.07 ± 5.725	93.87 ± 9.156	92.93 ± 4.559
MBP (mmHg)	99.2 ± 5.772	107.2 ± 9.697	104 ± 5.345	106.4 ± 4.852	103.7 ± 5.147
H.R. (bpm)	323.5 ± 29.05	223.3 ± 18.24^a^	278.4 ± 19.18^a^ ^,^ ^b^	264.2 ± 18.48^a^	307.8 ± 32.19^b^ ^,^ ^d^

Results are expressed as mean ± S.D. (N = 15) at *p* < 0.05 using ANOVA prior to Tukey’s *post hoc* test for multiple comparisons.

^a^ significant difference from the control group.

^b^ significant difference from the MET group.

^c^ significant difference from the Lina group.

^d^ significant difference from the SDG group.

Abbreviations: Hcy, homocysteine; Lina, linagliptin; SDG, secoisolariciresinol diglucoside; MET, methionine; HW/BW, heart weight to body weight ratio; SBP, systolic blood pressure; DBP, diastolic blood pressure; MBP, mean blood pressure; HR, heart rate.

Regarding body weight, SBP, DBP, and MBP, the five groups showed a non-significant difference (Table 1). However, the MET group showed a significant decrease in HR by 68.9% and an increase in HW and HW/BW by 1.6- and 1.5-fold, respectively, compared to that in the control group (Table 1). Pretreatment with Lina showed a significant increase in HR by 1.25-fold and a decrease in HW and HW/BW by 21.8% and 21.4%, respectively, compared to that in the MET group. However, pretreatment with SDG showed a significant increase in HR by 1.18-fold and a decrease in HW and HW/BW by 20.2% and 19.1%, respectively, compared to that in the MET group. Combined Lina + SDG showed a significant increase in HR by 1.38-fold and a decrease in HW and HW/BW by 30.17% and 25.8%, respectively, compared to that in the MET group.

**FIGURE 2:**
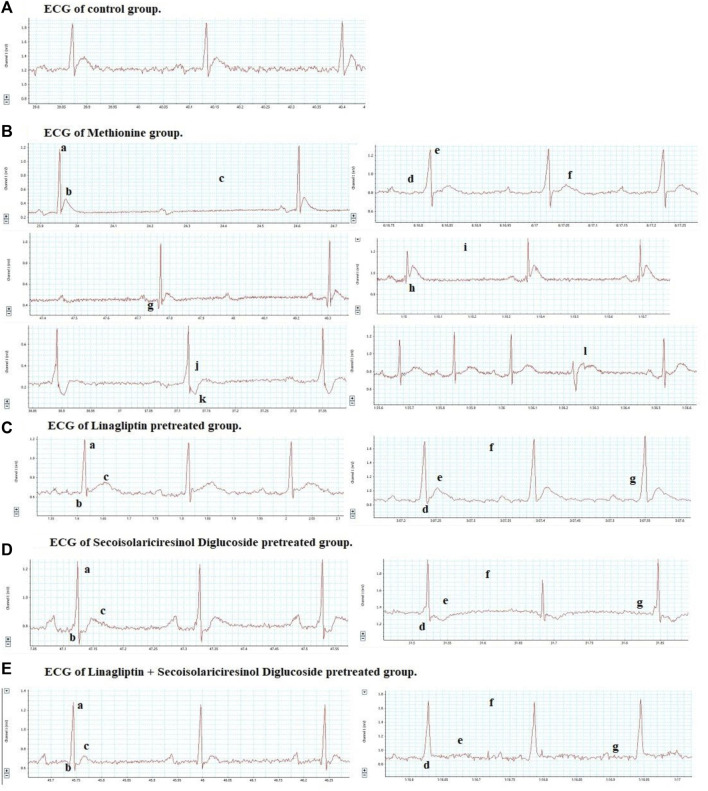
Effects of Lina (3 mg/kg/day. p.o.), SDG (20 mg/kg/day, p.o.), and their combination on ECG measurements in MET-treated rats. Representative ECG graphs of: **(A)** control group with a typical pattern. **(B)** MET group: a) elevated R-wave amplitude, b) elevated T-wave amplitude, c) dropped QRS-wave (second-degree heart block), d) increased PR interval (first-degree heart block), e) increased QRS duration, f) increased QT interval, g) pathological Q-wave, h) ST-segment elevation, i) increased RR interval, j) ST-segment depression, k) T-wave inversion, and l) lack of relation between P and R waves (third-degree heart block). **(C)** Lina-treated group: a) decreased R-wave amplitude, b) decreased Q-wave amplitude, c) decreased T-wave amplitude, d) decreased QRS-wave duration, e) decreased QT interval, f) decreased RR interval, and g) decreased PR interval. **(D)** SDG-treated group: a) decreased R-wave amplitude, b) decreased Q-wave amplitude, c) decreased T-wave amplitude, d) ST-segment depression, e) T-wave inversion, f) decreased RR interval, and g) decreased PR interval. **(E)** Lina + SDG-treated group: a) decreased R-wave amplitude, b) decreased Q-wave amplitude, c) decreased T-wave amplitude, d) decreased QRS-wave duration, e) decreased QT interval, f) decreased RR interval, and g) decreased PR interval. Abbreviations: Lina, linagliptin; SDG, secoisolariciresinol diglucoside; MET, methionine; ECG, electrocardiography.

ECG pattern changes were remarkably triggered by chronic administration of MET, as manifested by enhanced amplitudes of R, Q, and T waves; QRS-wave duration; QT interval; and RR interval by 1.5, 2.9, 3, 1.5, 1.7, and 2-fold, respectively, compared to that in the control group (Table 2; Figure 2). Moreover, the ECG of the MET group displayed ST-segment depression, ST-segment elevation, and T-wave inversion in 40%, 60%, and 40% of rats, respectively, compared to that in the control group. Additionally, as shown in the ECG, the following forms of atrioventricular (AV) blocks were detected: 30% of rats developed a first-degree AV block, 30% of rats developed a second-degree AV block, and 40% of rats developed a third-degree AV block in the MET group, as indicated by a prolonged PR interval, irregular P-wave to R-wave ratio, and a lack of relation between P and R waves, respectively.

**TABLE 2:** Effects of Lina (3 mg/kg/day. p.o.), SDG (20 mg/kg/day, p.o.), and their combination on the electrocardiographic (ECG) patterns in MET-treated rats.

Parameter	Control	MET	Lina	SDG	Lina + SDG
R-wave amplitude (mV)	0.5744 ± 0.02455	0.8831 ± 0.2099^a^	0.7012 ± 0.1335^b^	0.6967 ± 0.09513^b^	0.5826 ± 0.03054^b^
Q-wave amplitude (mV)	−0.021 ± 0.01187	−0.064 ± 0.02354^a^	−0.021 ± 0.01163^b^	−0.041 ± 0.01356^a^ ^,^ ^b^ ^,^ ^c^	−0.027 ± 0.00975^b^
T-wave amplitude (mV)	0.0661 ± 0.009538	0.1991 ± 0.01379^a^	0.1007 ± 0.01379^a^ ^,^ ^b^	0.1449 ± 0.04547^a^ ^,^ ^b^ ^,^ ^c^	0.0813 ± 0.005192^b^ ^,^ ^d^
QRS-wave duration (s)	0.01264 ± 0.001210	0.01863 ± 0.005081^a^	0.01467 ± 0.0011^a^ ^,^ ^b^	0.01573 ± 0.0005354^a^ ^,^ ^b^	0.01309 ± 0.0008523^b^ ^,^ ^c^ ^,^ ^d^
QT interval (s)	0.04084 ± 0.00348	0.07025 ± 0.004409^a^	0.05692 ± 0.0068^a^ ^,^ ^b^	0.0616 ± 0.007886^a^ ^,^ ^b^	0.04913 ± 0.003335^a^ ^,^ ^b^ ^,^ ^c^ ^,^ ^d^
RR interval (s)	0.1689 ± 0.03085	0.3391 ± 0.1127^a^	0.2033 ± 0.05148^b^	0.2211 ± 0.04044^b^	0.1888 ± 0.02282^b^

Results are expressed as mean ± S.D. (N = 15) at *p* < 0.05 using ANOVA prior to Tukey’s *post hoc* test for multiple comparisons.

^a^ significant difference from the control group.

^b^ significant difference from the MET group.

^c^ significant difference from the Lina group.

^d^ significant difference from the SDG group.

Abbreviations: Lina, linagliptin; SDG, secoisolariciresinol diglucoside; MET, methionine; mV, millivolt; s, second.

Pretreatment with Lina showed a significant improvement of the ECG pattern, as detected by the decreased amplitudes of R, Q, and T waves; QRS duration; QT interval; and RR interval by 18.2%, 4.7%, 9.9%, 0.4%, 1.3%, and 13.6%, respectively, compared to that in the MET group. Compared to the control group, rats treated with Lina displayed a non-significant difference in the ECG parameters except for the enhanced T-wave amplitude, QRS duration, and QT interval.

Moreover, pretreatment with SDG exhibited a significant improvement in the ECG pattern, as manifested by the decreased amplitudes of R, Q, and T waves; QRS duration; QT interval; and RR interval by 18.6%, 2.6%, 5.5%, 0.3%, 0.9%, and 11.8%, respectively, compared to that in the MET group. Additionally, the ECG of the SDG group displayed ST-segment depression in 60% of rats and T-wave inversion in 40% of rats. Compared to control rats, rats treated with SDG displayed a non-significant difference (*p* > 0.05), regarding the R-wave amplitude and RR interval.

Interestingly, combined Lina + SDG pretreatment led to a significant improvement of the ECG pattern, as indicated by the declined amplitudes of R, Q, and T waves; shortened QRS duration; and decreased QT and RR intervals by 30%, 4.3%, 11.9%, 0.6%, 2.11%, and 15.1%, respectively, compared to that in the MET group. The combined treatment group displayed a non-significant difference in the ECG parameters compared to control rats except for the QT interval.

### 3.2 Effects of Lina, SDG, and their combination on CK-MB, cTnI, and ANP levels

Cardiac damage and hypertrophy were further evaluated by measuring CK-MB, cTnI, and ANP levels. The MET group depicted a remarkable enhancement in CK-MB, cTnI, and ANP serum levels by 2.3, 3.6, and 3.7-fold, respectively, compared to that in the control group (Table 3). However, pretreatment with Lina showed a significant decrease in CK-MB, cTnI, and ANP serum levels by 40.3%, 50.7%, and 45%, respectively, compared to that in the MET group. Rats treated with SDG showed a significant decline of CK-MB, cTnI, and ANP serum levels by 38%, 48.6%, and 41.9%, respectively, compared to that in the MET group. Combined Lina + SDG pretreatment led to a significant decrease of CK-MB, cTnI, and ANP serum levels by 48.2%, 59.6%, and 55.9%, respectively, compared to that in the MET group (Table 3).

**TABLE 3:** Effects of Lina (3 mg/kg/day. p.o.), SDG (20 mg/kg/day, p.o.), and their combination on serum levels of cardiac CK-MB, cTnI, and ANP in MET-treated rats.

Parameter	Control	MET	Lina	SDG	Lina + SDG
CK-MB	118.5 ± 2.917	272.4 ± 12.75^a^	162.5 ± 13.32^a^ ^,^ ^b^	168.9 ± 7.078^a^ ^,^ ^b^	141.2 ± 5.615^a^ ^,^ ^b^ ^,^ ^c^ ^,^ ^d^
cTnI	84.35 ± 7.388	301.9 ± 25.23^a^	148.9 ± 11.64^a^ ^,^ ^b^	155.1 ± 14.17^a^ ^,^ ^b^	121.9 ± 10.91^a^ ^,^ ^b^ ^,^ ^c^ ^,^ ^d^
ANP	37.22 ± 1.015	138.1 ± 6.446^a^	75.97 ± 6.718^a^ ^,^ ^b^	80.28 ± 4.405^a^ ^,^ ^b^	60.92 ± 6.852^a^ ^,^ ^b^ ^,^ ^c^ ^,^ ^d^

Results are expressed as mean ± S.D. (N = 6) at *p* < 0.05 using ANOVA prior to Tukey’s post hoc test for multiple comparisons.

^a^ significant difference from control group.

^b^ significant difference from MET group.

^c^ significant difference from Lina group;

^d^ significant difference from SDG group.

Abbreviations: Lina, Linagliptin; SDG, Secoisolariciresinol Diglucoside; MET, Methionine; CK-MB, creatine kinase- MB; cTnI, cardiac troponin I; ANP, atrial natriuretic peptide.

### 3.3 Effects of Lina, SDG, and their combination on the lipid profile, atherogenic index, and atherogenic coefficient

**FIGURE 3:**
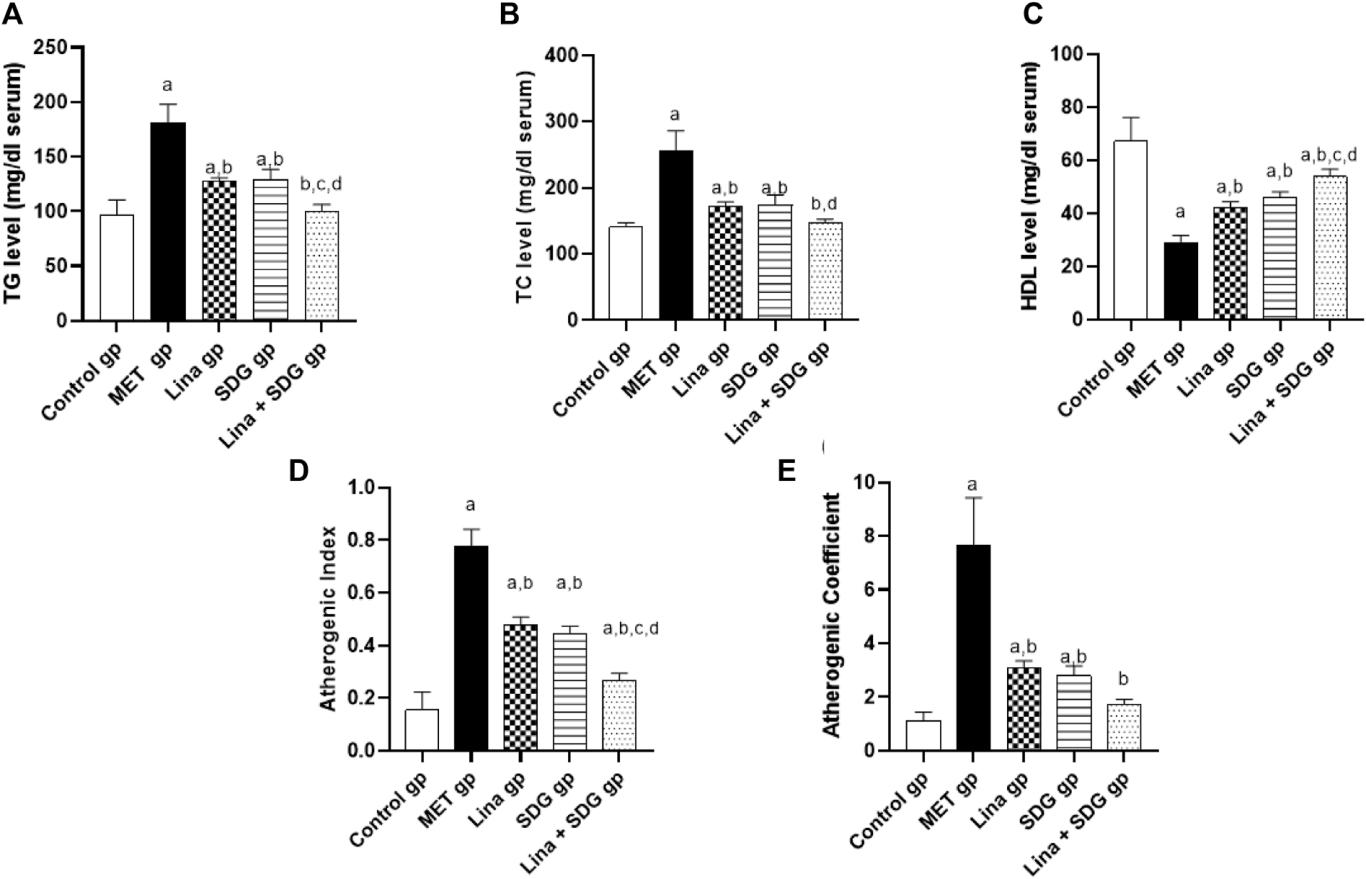
The effect of Lina (3 mg/kg/day. p.o.), SDG (20 mg/kg/day, p.o.) and their combination on the lipid profile, atherogenic index and atherogenic coefficient in MET -treated rats. **(A)** TG **(B)** TC **(C)** HDL **(D)** Atherogenic index and **(E)** Atherogenic coefficient. Results are expressed as mean ± S.D. (N = 6) at *p* = 0.05 using ANOVA prior to Tukey’s post hoc test for multiple comparisons. a, significant difference from control group; b, significant difference from MET group; c, significant difference from Lina group; d, significant difference from SDG group. Abbreviations: Lina, Linagliptin; SDG, Secoisolariciresinol Diglucoside; MET, Methionine; TG, Triglyceride; TC, Total cholesterol; HDL, High density lipoprotein.

The antihyperlipidemic effect of Lina, SDG, and their combination in MET-treated rats was evaluated by measuring TG, TC, and HDL serum levels. TG and TC serum levels were significantly elevated in the MET group by 1.9 and 1.8-fold, respectively, compared to those in the control group (Figures 3A, B). However, pretreatment with Lina, SDG, or combined Lina + SDG showed a significant reduction of the TG level by 29.6%, 28.7%, and 44.5%, respectively, and the TC level by 33%, 32%, and 42.6%, respectively, compared to those in the MET group (Figures 3A, B). Interestingly, pretreatment with Lina, SDG, or combined Lina + SDG showed a significant increase in the serum HDL level by 1.5, 1.6, and 1.9-fold, respectively, compared to that in the MET-a group, which showed a notable decline in serum HDL by 57.2% compared to that in the control group (Figure 3C).

Regarding atherogenic index, pretreatment with Lina, SDG, and combined Lina + SDG showed a significant decrease by 38.5%, 42.5%, and 65.4%, respectively, compared to that in the MET group, which showed a significant increase of the atherogenic index by 5-fold compared to that in the control group (Figure 3D). Regarding the atherogenic coefficient, pretreatment with Lina, SDG, or combined Lina + SDG showed a significant decrease by 59.9%, 63.6%, and 77.4%, respectively, compared to that in the MET group, which showed a notable enhancement of the atherogenic coefficient by 6.8-fold compared to that in the control group (Figure 3E).

### 3.4 Effects of Lina, SDG, and their combination on cardiac oxidative stress markers

**FIGURE 4:**
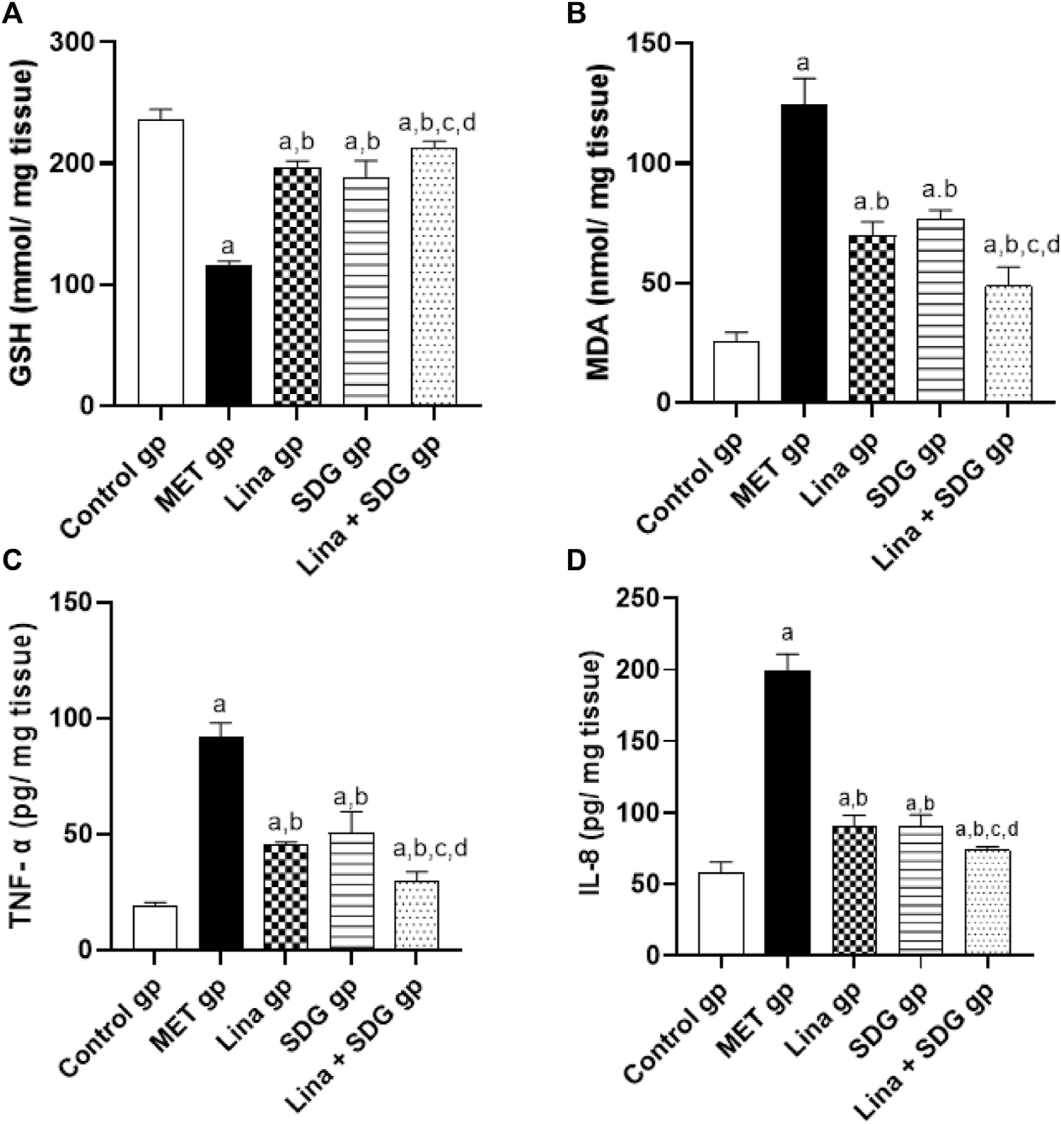
The effect of Lina (3 mg/kg/day. p.o.), SDG (20 mg/kg/day, p.o.) and their combination on cardiac oxidative stress and inflammatory markers in MET -treated rats. **(A)** GSH **(B)** MDA **(C)** TNF- α and **(D)** IL-8 levels. Results are expressed as mean ± S.D. (N = 6) at *p* < 0.05 using ANOVA prior to Tukey’s post hoc test for multiple comparisons. a, significant difference from control group; b, significant difference from MET group; c, significant difference from Lina group; d, significant difference from SDG group. Abbreviations: Lina, Linagliptin; SDG, Secoisolariciresinol Diglucoside; MET, Methionine; GSH, Glutathione; MDA, Malondialdehyde; TNF- α, Tumor necrosis factor- alpha; IL-8, Interlukin-8.

GSH and MDA levels in heart tissue were measured to evaluate cardiac antioxidant activity and lipid peroxidation. The MET group elucidated a remarkable decrease in the cardiac GSH level by 50.6% and a notable augmentation in the MDA level by 4.8-fold compared to the control group (Figures 4A, B). Contrarily, pretreatment with Lina, SDG, and combined Lina + SDG showed a significant rise in the GSH level by 1.7, 1.6, and 1.9-fold, respectively (Figure 4A), and a significant decrease in the MDA level by 44%, 38.2%, and 60.9%, respectively, compared to that in the MET group (Figure 4B).

### 3.5 Effects of Lina, SDG, and their combination on cardiac inflammatory markers

To examine the anti-inflammatory effect of Lina and SDG, TNF-α and IL-8 levels were measured (Figures 4C, D). The MET group depicted a significant upregulation in TNF-α and IL-8 levels by 4.7 and 3.4-fold, respectively, compared to the control group (Figures 4C, D). However, pretreatment with Lina, SDG, and combined Lina + SDG showed a significant decrease in TNF-α by 50.7%, 45.1%, and 67.9%, respectively (Figure 4C), and the IL-8 level by 55.1%, 54.6%, and 63.2%, respectively, compared to that in the MET group (Figure 4D).

### 3.6 Effects of Lina, SDG, and their combination on cardiac caspase-3

**FIGURE 5:**
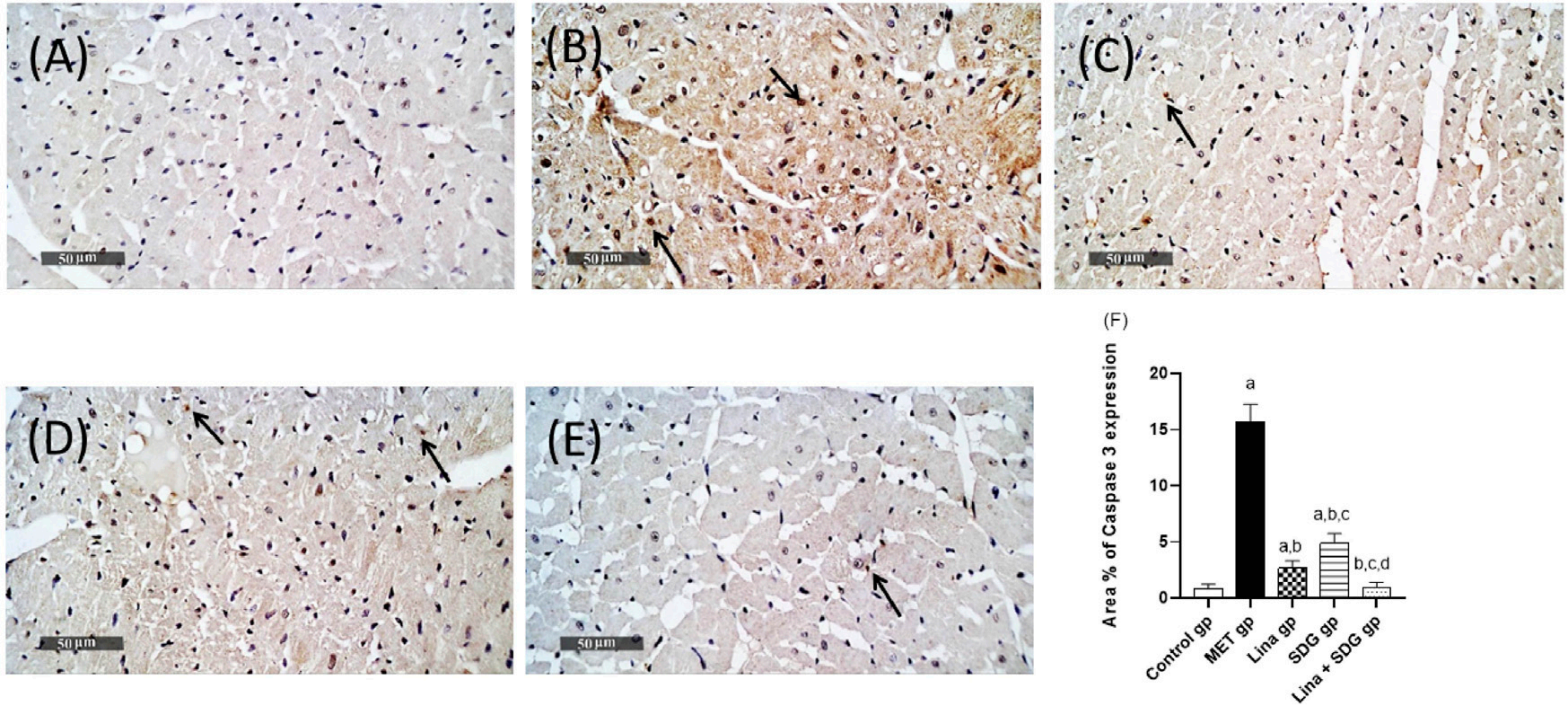
Effects of Lina (3 mg/kg/day. p.o.), SDG (20 mg/kg/day, p.o.), and their combination on caspase-3 expression levels in MET-treated rats. **(A)** Control group, **(B)** MET group, **(C)** Lina-treated group, **(D)** SDG-treated group, **(E)** Lina + SDG-treated group, and **(F)** mean area percentage of expression of caspase-3 (scale bar = 50 µm). Results are expressed as mean ± S.D. (N = 6) at *p* < 0.05 using ANOVA prior to Tukey’s *post hoc* test for multiple comparisons. a: Significant difference from the control group; b: significant difference from the MET group; c: significant difference from the Lina group; d: significant difference from the SDG group. Abbreviations: Lina, linagliptin; SDG, secoisolariciresinol diglucoside; MET, methionine.

Cardiac tissues of the MET group showed a notable protein expression of the proapoptotic marker caspase-3. On the contrary, pretreatment with Lina, SDG, and their combination reduced the expression of caspase-3 by 83%, 69%, and 93.7%, respectively, compared to that in the MET group (Figure 5).

### 3.7 Effects of Lina, SDG, and their combination on ER stress markers, GRP78, PERK, ATF4, CHOP, and SREBP1c

**FIGURE 6:**
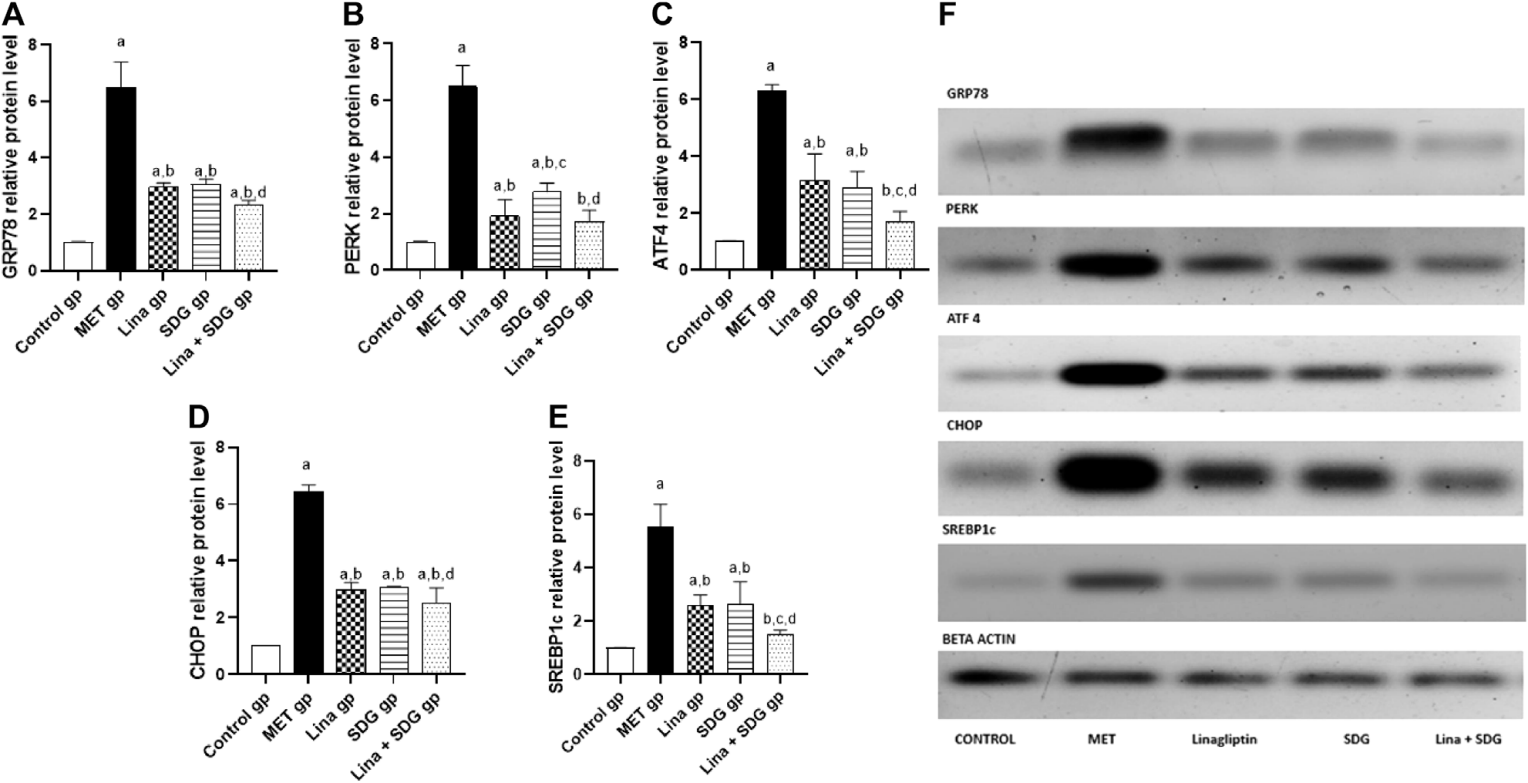
The effect of Lina (3 mg/kg/day. p.o.), SDG (20 mg/kg/day, p.o.) and their combination on cardiac expression of **(A)** GRP78, **(B)** PERK, **(C)** ATF4, **(D)** CHOP, and **(E)** SREBP1c in relation to β-actin. Results are expressed as mean ± S.D. (N = 6) at *p* < 0.05 using ANOVA prior to Tukey’s *post hoc* test for multiple comparisons. a: significant difference from control group; b: significant difference from MET group; c: significant difference from Lina group; d: significant difference from SDG group. **(F)** Representative western blot bands showing the expression of GRP78, PERK, ATF4, CHOP, SREBP1c and β-actin in all groups. Abbreviations: Lina: Linagliptin, SDG: Secoisolariciresinol Diglucoside, MET: Methionine, GRP78: glucose- regulated protein 78, PERK: Protein kinase RNA- like Endoplasmic reticulum kinase, ATF4: activating transcription factor 4, CHOP: C/EBP homologous protein, SERBP1c: sterol regulatory element-binding protein 1c.

As elucidated in Figure 6, chronic administration of MET led to a remarkable increase in the protein expression of GRP78, PERK, ATF4, CHOP, and SREBP1c in cardiac tissues by 6.4, 6.4, 6.2, 6.4, and 5.5-fold, respectively, in comparison with that in the control group. On the other hand, pretreatment with Lina resulted in a notable inhibition of GRP78, PERK, ATF4, CHOP, and SREBP1c expression by 54.1%, 70.4%, 49.6%, 53.7%, and 53.2%, respectively, compared to that in the MET group. Moreover, pretreatment with SDG showed a significant decrease in GRP78, PERK, ATF4, CHOP, and SREBP1c expression by 52.7%, 57.3%, 54%, 52.4%, and 53%, respectively, compared to that in the MET group. Pretreatment with combined Lina + SDG showed a remarkable decline of GRP78, PERK, ATF4, CHOP, and SREBP1c expression by 64%, 73.5%, 73%, 60.8%, and 73.4%, respectively, compared to that in the MET group.

### 3.8 Effects of Lina, SDG, and their combination on histopathological features

**FIGURE 7:**
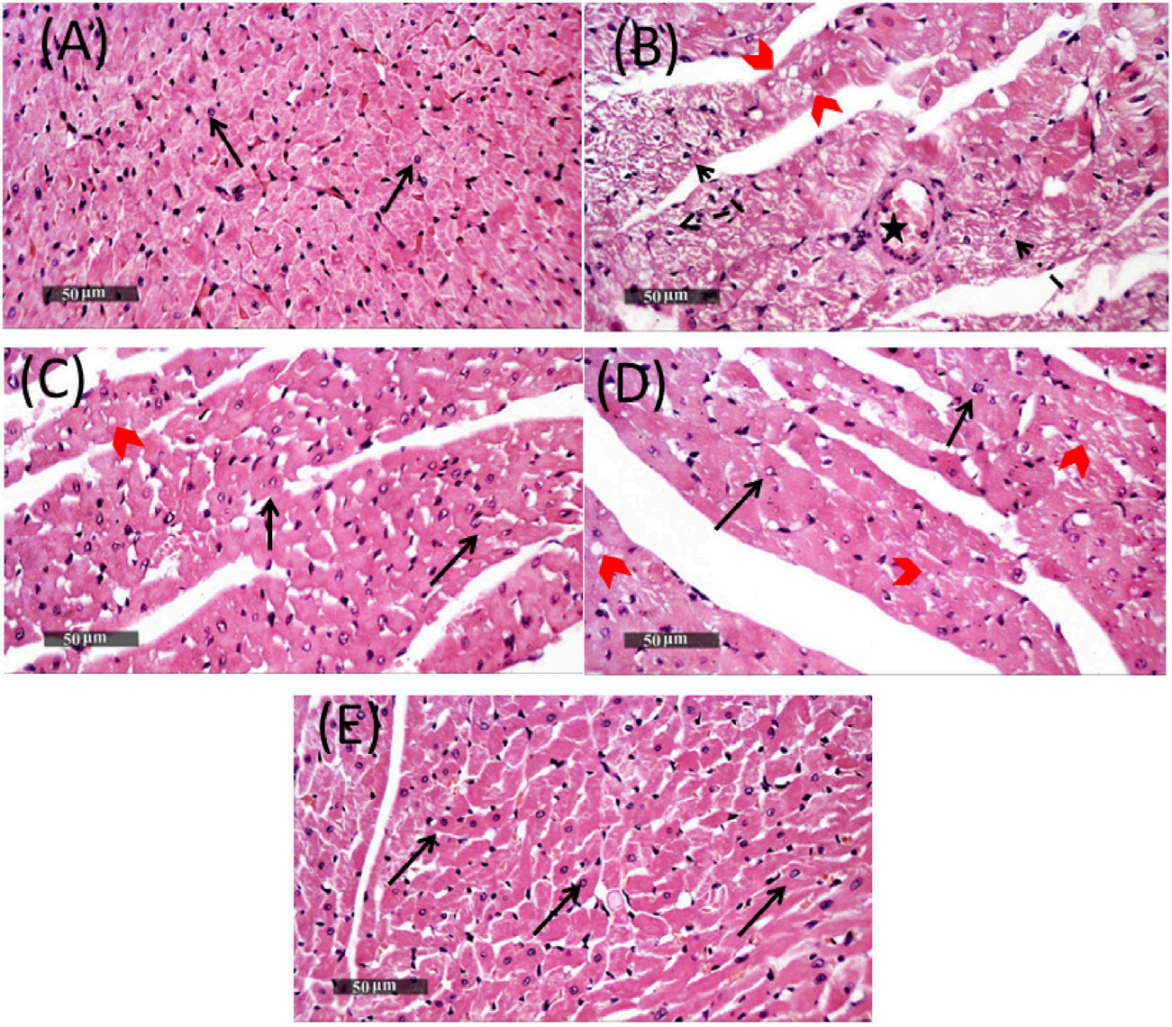
Representative photomicrographs of heart tissues stained with H&E, ×400 magnification, scale bar = 50 µm, showing the effect of Lina (3 mg/kg/day. p.o.), SDG (20 mg/kg/day, p.o.), and their combination on MET-treated rats. **(A)** Cardiac tissue section of the control group demonstrating normal organized histological features of cardiac walls with intact endocardium, myocardium, and pericardial layers with apparent intact branched cardiomyocytes showing intact visible nuclear and subcellular details **(arrow)**. Normal interstitial tissues with intact vasculatures are shown. **(B)** Cardiac tissue section of the MET group showing focal areas of vacuolar and fatty changes of cardiomyocytes **(red arrow head)** with alternated figures of fragmented myofibrils in some muscle fibers with pyknotic nuclei **(dashed arrow)** and apparent intact cardiomyocytes. Mild-to-moderate records of congested **(B)** BVs were observed **(star)**. **(C)** Cardiac tissue section of the Lina-treated group showing minimal records of vacuolar and fatty changes of cardiomyocytes all over examined tissue sections (***red arrow head***) with almost apparent intact cellular details (***black arrow***), as well as interstitial tissue. **(D)** Cardiac tissue section of the SDG-treated group showed almost intact histological features in most of examined tissue sections (***black arrow***) with occasional mild records of focal degenerated cardiomyocytes (***red arrow head***). **(E)** Cardiac tissue section of the Lina + SDG-treated group showed well-organized histological features of the cardiac wall resembling normal controls. Abbreviations: Lina, linagliptin; SDG, secoisolariciresinol diglucoside; MET, methionine; H&E, hematoxylin and eosin.

Cardiac tissue sections stained with H&E of the MET group showed focal areas of vacuolar and fatty changes of cardiomyocytes with alternated figures of fragmented myofibrils in some muscle fibers with pyknotic nuclei and apparent intact cardiomyocytes. Mild-to-moderate records of congested blood vessels were also observed (Figure 7). On the other hand, cardiac tissue sections obtained from the Lina-treated group showed minimal records of vacuolar and fatty changes of cardiomyocytes throughout the examined tissue sections, with almost apparent intact cellular details and interstitial tissue. In addition, pretreatment with SDG revealed almost intact histological features in most of the examined cardiac tissue sections, with occasional mild records of focal degenerated cardiomyocytes. Pretreatment with combined Lina + SDG showed well-organized histological features of the cardiac wall comparable to that of the normal control (Figure 7).

## 4 Discussion

Our study was designed to establish the effect of HHcy on the cardiac tissue and to assess the antihyperlipidemic and cardiac improvement effects of Lina, SDG, and their combination on MET-treated rats. Our results showed a significant increase in Hcy, TC, and TG serum levels and a remarkable decline in HDL serum levels in MET-treated rats compared to that in the control group. Previous studies revealed that increased serum levels of Hcy stimulate SREBP-1 and SREBP-2, which regulate genes responsible for triglyceride biosynthesis and cholesterol production, leading to accelerated LDL oxidation (Werstuck et al., 2001; Woo et al., 2005). Furthermore, previous research has shown that Hcy lowers HDL-C levels in plasma by inhibiting the hepatic synthesis of apo A-I, the primary HDL apolipoprotein (Barter and Rye, 2006).

Moreover, apart from its effect on coronary blood flow, hyperlipidemia contributes to various direct processes that impact the ultrastructure of the myocardium, such as inflammation and the oxidative stress state, fibrosis, and inadequate autophagy, leading to heart damage (Yao et al., 2020).

Additionally, hemodynamic changes caused by HHcy were evaluated, revealing a significantly reduced heart rate (HR). These results followed previous studies (Kennedy et al., 2006; Study, 2021; Joseph et al., 2022). Moreover, ECG records of the MET-treated rats indicated a significant increase in R-wave and T-wave amplitudes, QRS duration, QT interval, and RR interval compared to that in the control group. In the same context, the MET group displayed ST-segment depression, ST-segment elevation, T-wave inversion, and forms of AV blocks. A large R-wave indicates an enlarged left ventricle mass, whereas prolonged QRS and QT intervals indicate a larger ventricular mass taking longer to depolarize and repolarize m (Sysa-Shah et al., 2015; Zipes et al., 2019). In the same context, persistent T-wave inversion and ST-segment elevation are indicators of myocardial infarction (Coppola et al., 2013), while ST-segment depression indicates myocardial ischemia (Al-Zahrani et al., 2015). Furthermore, ECG showed the three types of AV blocks (Carll et al., 2013; Da Cunha et al., 2014; Chowdhury et al., 2015).

Myocardial damage and infarction were confirmed by measuring cTnI and CK-MB levels (Cao et al., 2021), whereas the ANP level was estimated as an indicator for hypertrophied hearts (Hayashi et al., 2004). Serum levels of cTnI, CK-MB, ANP, and HW/BW were significantly elevated in MET-treated rats. Previous studies showed that HHcy causes cardiac injury and remodeling through several mechanisms, such as the myocardial collagen buildup, fibrosis, systolic dysfunction, and the activated myocardial redox state (Weiss et al., 2001; Raaf et al., 2011).

The role of ER stress in the pathophysiology of many diseases is indisputable, which prompted us to study the effect of HHcy on the ER stress as a contributing pathway to the development of heart dysfunction. HHcy exacerbates the myocardial cellular redox state by the highly reactive thiol group of Hcy, which undergoes an autoxidation reaction, decreasing the expression and activities of antioxidants, resulting in ROS generation (Weiss et al., 2001). Accordingly, HHcy-induced oxidative stress elicits ER stress, which triggers GRP78 protein expression, which, in turn, activates the PERK and IRE-1 pathways (Colgan et al., 2007). Activation of PERK upregulates the expression of ATF-4, leading to the generation of ROS, activation of NF-κB, and CHOP protein, a promotor of apoptosis (Zhang and Kaufman, 2008). In addition, ER stress activates SREBP expression, leading to increase in intracellular lipid levels and cardiac lipidopathy, which causes mitochondrial dysfunction, which finally leads to ventricular fibrosis and malfunction (Puukila et al., 2017; Study, 2021). In our study, the protein expression of GRP78, PERK, ATF-4, NF-κB, CHOP, and SREBP1c was remarkably enhanced in the MET-treated group than in the control group, granting a decisive confirmation of the involvement of ER stress in the pathophysiology of HHcy.

Moreover, these previously mentioned data conform with our findings, wherein cardiac levels of MDA, TNF-α, IL-8, and apoptotic marker caspase-3 were significantly increased, while the GSH level was notably reduced in MET-treated rats.

Our research was further extended to study the antihyperlipidemic and cardioprotective effects of Lina, SDG, and their combination on MET-treated rats. According to our findings, Lina, SDG, and their combination decreased the Hcy serum level and notably improved the lipid profile. A previous study revealed that DPP-4 inhibitors decrease the Hcy serum level by enhancing the storage level of vitamin B12, which is essential for Hcy metabolism (Tammen et al., 2023). Moreover, the mechanism by which SDG can decrease the Hcy level is unclear. However, Jose et al. (2018) suggested that oxidative stress decreases the folate level, and accordingly, due to its antioxidant properties, SDG may increase the folate level, which is critically involved in Hcy metabolism. Additionally, it was found that pretreatment with either Lina or SDG resulted in an improvement of cardiac injury and hypertrophy, as evidenced by the significant decrease of HW/BW, improved ECG pattern, and reduced serum levels of cardiac markers, indicating the protective role of Lina and SDG against cardiac ischemia/infarction and ventricular hypertrophy compared to MET-treated rats. Moreover, both Lina and SDG remarkably reduced oxidative stress, and inflammatory and apoptotic markers compared to the MET-treated group. These results follow previous studies that reported the antioxidant and anti-inflammatory effects of Lina and SDG (Santos et al., 2020; Wei et al., 2020).

In the present study, both Lina and SDG remarkably downregulated ER stress biomarkers compared to the MET-treated group. The role of Lina on ER stress in the heart was investigated based on studies applied to the liver and brain of rats, which showed a significant decrease in ER stress biomarkers when treated with Lina (El-Deeb et al., 2020; Santos et al., 2020). In the same context, according to a previous study (Wei et al., 2020), SDG significantly alleviated the enhanced mRNA expression of ER stress mediators in high-fat and high-fructose diet (HFFD)-induced hepatic lipid metabolic disorders.

Finally, our histopathological examination of the cardiac tissues further confirmed the aforementioned findings.

It is worth mentioning that the improvement produced due to the combined administration of Lina and SDG remarkably surpassed the improvement predisposed by the intake of Lina and SDG separately to the extent that the lipid profile, ECG pattern serum levels of cardiac markers, and the histopathological observations obtained from the combined Lina + SDG group were comparable to those of the control group.

## 5 Conclusion

In the current study, we examined the cardioprotective effects of Lina, SDG, and their combination against HHcy-induced hyperlipidemia and cardiac hypertrophy in rats. The findings of this investigation asserted the cardioprotective effect of Lina and SDG against HHcy-induced hyperlipidemia and cardiac dysfunction in rats through the inhibition of the ER stress pathway with the consequent antioxidant, anti-inflammatory, and anti-apoptotic effects.

## 6 Limitations to the study

A major limitation to all the dietary models of hyperhomocysteinemia is that elevation of homocysteine is almost always accompanied by alterations in other metabolites that may influence vascular pathophysiology (Dayal and Lentz, 2008). In addition, the most common cause of HHcy is thought to be enzyme abnormalities related to Hcy metabolism rather than methionine overconsumption. Finally, although the study results are of interest, extrapolating these findings to humans would have positively influenced the study.

## Data Availability

The raw data supporting the conclusion of this article will be made available by the authors, without undue reservation.

## Abbreviations

SDG, secoisolariciresinol diglucoside; GRP78, glucose-regulated protein 78; SREBPs, sterol regulatory element-binding proteins; IRE1, inositol-requiring enzyme 1; PERK, protein kinase RNA- like endoplasmic reticulum kinase; ATF 6, activating transcription factor 6; CHOP, C/EBP homologous protein; NF-kB, nuclear factor kappa-b; TNF-α, tumor necrosis factor-alpha; IL 8, interleukin-8.

## References

[B1] Al-Zahrani Y. A. Al-Harthi S. E. Khan L. M. El-Bassossy H. M. Edris S. M. Sattar M. A. A. A. (2015). The possible antianginal effect of allopurinol in vasopressin-induced ischemic model in rats. Saudi Pharm. J. 23 (5), 487–498. 10.1016/J.JSPS.2014.12.001 26594114 PMC4605908

[B2] Ambrosi N. Arrosagaray V. Guerrieri D. Uva P. D. Petroni J. Herrera M. B. (2016). α-Lipoic acid protects against ischemia-reperfusion injury in simultaneous kidney-pancreas transplantation. Transplantation 100 (4), 908–915. 10.1097/TP.0000000000000981 26502371

[B3] Aykan D. A. Yaman S. Eser N. Özcan Metin T. Seyithanoğlu M. Aykan A. Ç. (2019). Bisoprolol and linagliptin ameliorated electrical and mechanical isometric myocardial contractions in doxorubicin-induced cardiomyopathy in rats. Pharmacol. Rep. 72 (4), 867–876. 10.1007/S43440-019-00034-9 32048248

[B4] Ayyappan J. P. Lizardo K. Wang S. Yurkow E. Nagajyothi J. F. (2020). Inhibition of SREBP improves cardiac lipidopathy, improves endoplasmic reticulum stress, and modulates chronic chagas cardiomyopathy. J. Am. Heart Assoc. 9 (3), e014255. 10.1161/JAHA.119.014255 31973605 PMC7033903

[B5] Barter P. J. Rye K.-A. (2006). Homocysteine and cardiovascular disease. Circulation Res. 99 (6), 565–566. 10.1161/01.RES.0000243583.39694.1f 16973911

[B54] Bonaventura D. Tirapelli C. R. De Oliveira A. M. (2009). Chronic methionine load-induced hyperhomocysteinemia impairs the relaxation induced by bradykinin in the isolated rat carotid. Amino Acids, 37 (4). 617–627. 10.1007/S00726-008-0181-Z 18821053

[B6] Cao F. Wu K. Zhu Y. Z. Bao Z. W. (2021). Roles and mechanisms of dipeptidyl peptidase 4 inhibitors in vascular aging. Front. Endocrinol. 12, 958. 10.3389/fendo.2021.731273 PMC8416540 34489872

[B7] Carll A. P. Lust R. M. Hazari M. S. Perez C. M. Krantz Q. T. King C. J. (2013). Diesel exhaust inhalation increases cardiac output, bradyarrhythmias, and parasympathetic tone in aged heart failure - prone rats. Toxicol. Sci. 131 (2), 583–595. 10.1093/TOXSCI/KFS295 23047911 PMC3937610

[B8] Chowdhury R. Ashraf H. Melanson M. Tanada Y. Nguyen M. Silberbach M. (2015). Mouse model of human congenital heart disease: progressive atrioventricular block induced by a heterozygous nkx2-5 homeodomain missense mutation. Circulation Arrhythmia Electrophysiol. 8 (5), 1255–1264. 10.1161/CIRCEP.115.002720 PMC4618020 26226998

[B9] Chwatko G. Boers G. H. J. Strauss K. A. Shih D. M. Jakubowski H. (2007). Mutations in methylenetetrahydrofolate reductase or cystathionine beta-synthase gene, or a high-methionine diet, increase homocysteine thiolactone levels in humans and mice. FASEB J. 21 (8), 1707–1713. 10.1096/fj.06-7435com 17327360

[B10] Colgan S. M. Tang D. Werstuck G. H. Austin R. C. (2007). Endoplasmic reticulum stress causes the activation of sterol regulatory element binding protein-2. Int. J. Biochem. Cell Biol. 39 (10), 1843–1851. 10.1016/j.biocel.2007.05.002 17604677

[B11] Coppola G. Carità P. Corrado E. Borrelli A. Rotolo A. Guglielmo M. (2013). ST segment elevations: always a marker of acute myocardial infarction? Indian Heart J. 65 (4), 412–423. 10.1016/J.IHJ.2013.06.013 23993002 PMC3860734

[B12] Cuijpers I. Papageorgiou A. P. Carai P. Herwig M. Mügge A. Klein T. (2021). Linagliptin prevents left ventricular stiffening by reducing titin cleavage and hypophosphorylation. J. Cell. Mol. Med. 25 (2), 729–741. 10.1111/JCMM.16122 33295687 PMC7812306

[B55] Culling C. F. A. Charles F. A. (1974). Handbook of histopathological and histochemical techniques: (including museum techniques). 712.

[B13] Da Cunha D. N. Q. Pereira V. G. Favarato L. S. C. Okano B. S. Daibert A. P. F. Monteiro B. S. (2014). Acute and chronic observations of complete atrioventricular block in rats. Lab. Anim. 48 (3), 237–249. 10.1177/0023677214530905 24759570

[B14] Dayal S. Lentz S. R. (2008). Murine models of hyperhomocysteinemia and their vascular phenotypes. Arteriosclerosis, Thrombosis, Vasc. Biol. 28 (9), 1596–1605. 10.1161/ATVBAHA.108.166421 PMC2574668 18556571

[B15] Dorotea D. Koya D. Ha H. (2020). Recent insights into SREBP as a direct mediator of kidney fibrosis via lipid-independent pathways. Front. Pharmacol. 11, 265. 10.3389/fphar.2020.00265 32256356 PMC7092724

[B16] Elsayed H. E. Ebrahim H. Y. Mady M. S. Khattab M. A. El-Sayed E. K. Moharram F. A. (2022). Ethnopharmacological impact of Melaleuca rugulosa (Link) Craven leaves extract on liver inflammation. J. Ethnopharmacol. 292, 115215. 10.1016/j.jep.2022.115215 35337921

[B17] El‐Deeb O. S. Soliman G. M. Elesawy R. O. (2020). Linagliptin, the dipeptidyl peptidase‐4 enzyme inhibitor, lessens CHOP and GRP78 biomarkers levels in cisplatin‐induced neurobehavioral deficits: a possible restorative gateway. J. Biochem. Mol. Toxicol. 34 (9), e22541. 10.1002/jbt.22541 32567747

[B18] Francula-Zaninovic S. Nola I. A. (2018). Management of measurable variable cardiovascular disease’ risk factors. Curr. Cardiol. Rev. 14 (3), 153–163. 10.2174/1573403X14666180222102312 29473518 PMC6131408

[B19] Hayashi D. Kudoh S. Shiojima I. Zou Y. Harada K. Shimoyama M. (2004). Atrial natriuretic peptide inhibits cardiomyocyte hypertrophy through mitogen-activated protein kinase phosphatase-1. Biochem. Biophysical Res. Commun. 322 (1), 310–319. 10.1016/j.bbrc.2004.07.119 15313208

[B20] Jose S. Bhalla P. Suraishkumar G. K. (2018). Oxidative stress decreases the redox ratio and folate content in the gut microbe, Enterococcus durans (MTCC 3031). Sci. Rep. 8 (1), 12138. 10.1038/s41598-018-30691-4 30108274 PMC6092354

[B21] Joseph J. Joseph L. Shekhawat N. S. Devi S. Wang J. Melchert R. B. (2022). Hyperhomocysteinemia leads to pathological ventricular hypertrophy in normotensive rats. Am. J. Physiol. Heart Circ. Physiol. 72205, 679–686. 10.1152/ajpheart.00145.2003 12730062

[B22] Karaca M. Magnan C. Kargar C. (2009). Functional pancreatic beta-cell mass: involvement in type 2 diabetes and therapeutic intervention. Diabetes & Metabolism 35 (2), 77–84. 10.1016/j.diabet.2008.09.007 19251449

[B23] Kennedy R. H. Melchert R. B. Joseph J. (2006). Of hyperhomocysteinemia in Conscious Unrestrained rats, 94–97. 10.1016/j.amjhyper.2005.07.008 16461198

[B24] Kim H. C. (2021). Epidemiology of cardiovascular disease and its risk factors in Korea. Glob. Health & Med. 3 (3), 134–141. 10.35772/ghm.2021.01008 34250288 PMC8239378

[B25] Kim J. Kim H. Roh H. Kwon Y. (2018). Causes of hyperhomocysteinemia and its pathological significance. Archives Pharmacal Res. 41 (4), 372–383. 10.1007/s12272-018-1016-4 29552692

[B26] Koibuchi N. Hasegawa Y. Katayama T. Toyama K. Uekawa K. Sueta D. (2014). DPP-4 inhibitor linagliptin ameliorates cardiovascular injury in salt-sensitive hypertensive rats independently of blood glucose and blood pressure. Cardiovasc. Diabetol. 29 (13), 157. 10.1186/s12933-014-0157-0 PMC4255443 25471116

[B27] Liu B. Ma S. Wang T. Zhao C. Li Y. Yin J. (2016). A novel rat model of heart failure induced by high methionine diet showing evidence of association between hyperhomocysteinemia and activation of NF-kappaB. Am. J. Transl. Res. 8 (1), 117–124. 27069545 PMC4759421

[B28] Majors A. Ehrhart L. A. Pezacka E. H. (1997). Homocysteine as a risk factor for vascular disease. Enhanced collagen production and accumulation by smooth muscle cells. Arteriosclerosis, Thrombosis, Vasc. Biol. 17 (10), 2074–2081. 10.1161/01.ATV.17.10.2074 9351374

[B29] Mishra P. K. Tyagi N. Kundu S. Tyagi S. C. (2009). MicroRNAs are involved in homocysteine-induced cardiac remodeling. Cell Biochem. Biophysics 55 (3), 153–162. 10.1007/s12013-009-9063-6 PMC2863043 19669742

[B30] Outinen P. A. Sood S. K. Liaw P. C. Y. Sarge K. D. Maeda N. Hirsh J. (1998). Characterization of the stress-inducing effects of homocysteine. Biochem. J. 332 (1), 213–221. 10.1042/bj3320213 9576870 PMC1219470

[B31] Ozcan L. Tabas I. (2012). Role of endoplasmic reticulum stress in metabolic disease and other disorders. Annu. Rev. Med. 63, 317–328. 10.1146/annurev-med-043010-144749 22248326 PMC3290993

[B32] Puukila S. Oliveira R. Türck P. Campos C. Hellen J. Bonetto P. (2017). Secoisolariciresinol diglucoside attenuates cardiac hypertrophy and oxidative stress in monocrotaline-induced right heart dysfunction. Mol. Cell. Biochem. 432 (1), 33–39. 10.1007/s11010-017-2995-z 28321539

[B33] Qi J. Tan Y. Fan D. Pan W. Yu J. Xu W. (2020). Songling Xuemaikang Capsule inhibits isoproterenol-induced cardiac hypertrophy via CaMKIIδ and ERK1/2 pathways. J. Ethnopharmacol. 253, 112660. 10.1016/j.jep.2020.112660 32061912

[B34] Raaf L. Noll C. Cherifi M. E. H. Samuel J.-L. Delcayre C. Delabar J.-M. (2011). Myocardial fibrosis and TGFB expression in hyperhomocysteinemic rats. Mol. Cell. Biochem. 347 (1–2), 63–70. 10.1007/s11010-010-0612-5 20938722

[B35] Racek J. Rusnáková H. Trefil L. Siala K. K. (2005). The influence of folate and antioxidants on homocysteine levels and oxidative stress in patients with hyperlipidemia and hyperhomocysteinemia. Physiological Res. 54 (1), 87–95. 10.33549/physiolres.930520 15717846

[B56] Rehman T. Shabbir M. A. Inam‐Ur‐Raheem M. Manzoor M. F. Ahmad N. Liu Z. (2020). Cysteine and homocysteine as biomarker of various diseases. Food Sci. Nutr, 8 (9). 4696–4707. 10.1002/fsn3.1818 32994931 PMC7500767

[B36] Rom S. Zuluaga-Ramirez V. Reichenbach N. L. Erickson M. A. Winfield M. Gajghate S. (2018). Secoisolariciresinol diglucoside is a blood-brain barrier protective and anti-inflammatory agent: implications for neuroinflammation. J. Neuroinflammation 15 (1), 25. 10.1186/s12974-018-1065-0 29373982 PMC5787274

[B37] Santos F. O. Correia B. R. O. Marinho T. S. Brabosa-da-Silva S. Mandarim-de-Lacerda C. A. Souza-Mello V. (2020). Anti-steatotic linagliptin pleiotropic effects encompasses suppression of *de novo* lipogenesis and ER stress in high-fat-fed mice. Mol. Cell. Endocrinol. 509, 110804. 10.1016/J.MCE.2020.110804 32259637

[B38] Selhub J. Jacques P. F. Rush D. Rosenberg I. H. Wilson P. W. F. (1993). Vitamin status and intake as primary determinants of homocysteinemia in an elderly population. JAMA 270 (22), 2693–2698. 10.1001/JAMA.1993.03510220049033 8133587

[B39] Si J. Meng R. Gao P. Hui F. Li Y. Liu X. (2019). Linagliptin protects rat carotid artery from balloon injury and activates the NRF2 antioxidant pathway. Exp. Anim. 68 (1), 81–90. 10.1538/expanim.18-0089 30369549 PMC6389508

[B40] Singh M. Pathak M. S. Paul A. (2015). A study on atherogenic indices of pregnancy induced hypertension patients as compared to normal pregnant women. J. Clin. DIAGNOSTIC Res. 9, BC05–BC08. 10.7860/JCDR/2015/13505.6241 PMC4572947 26393117

[B41] Study E. Zhang W. Wang G. Zhao X. Gao N. Liu Z. (2021). Low dose of folic acid can ameliorate hyperhomocysteinemia-induced cardiac fibrosis and diastolic dysfunction in spontaneously hypertensive rats. Int. Heart J. 62, 627–635. 10.1536/ihj.20-593 33994505

[B42] Sysa-Shah P. Sørensen L. L. Roselle Abraham M. Gabrielson K. L. (2015). Electrocardiographic characterization of cardiac hypertrophy in mice that overexpress the ErbB2 receptor tyrosine kinase. Comp. Med. 65 (4), 295–307. 26310459 PMC4549675

[B43] Tammen H. Kömhoff M. Delić D. Lund S. S. Hocher B. Frankenreiter S. (2023). Linagliptin treatment is associated with altered cobalamin (VitB12) homeostasis in mice and humans. Sci. Rep. 13 (1), 601. 10.1038/s41598-023-27648-7 36635409 PMC9837112

[B44] Wang M. Wey S. Zhang Y. Ye R. Lee A. S. (2009). Role of the unfolded protein response regulator GRP78/BiP in development, cancer, and neurological disorders. Antioxidants Redox Signal. 11 (9), 2307–2316. 10.1089/ARS.2009.2485 PMC2819800 19309259

[B45] Wei L. Zhao C. Dong S. Yao S. Ji B. Zhao B. (2020). Secoisolariciresinol diglucoside alleviates hepatic lipid metabolic misalignment involving the endoplasmic reticulum-mitochondrial axis. Food Funct. 11 (5), 3952–3963. 10.1039/d0fo00124d 32426795

[B46] Weiss N. Zhang Y. Y. Heydrick S. Bierl C. Loscalzo J. (2001). Overexpression of cellular glutathione peroxidase rescues homocyst(e)ine-induced endothelial dysfunction. Proc. Natl. Acad. Sci. U. S. A. 98 (22), 12503–12508. 10.1073/pnas.231428998 11606774 PMC60083

[B47] Werstuck G. H. Lentz S. R. Dayal S. Hossain G. S. Sood S. K. Shi Y. Y. (2001). Homocysteine-induced endoplasmic reticulum stress causes dysregulation of the cholesterol and triglyceride biosynthetic pathways. J. Clin. Investigation 107 (10), 1263–1273. 10.1172/JCI11596 PMC209295 11375416

[B48] Woo C. W. H. Siow Y. L. Pierce G. N. Choy P. C. Minuk G. Y. Mymin D. (2005). Hyperhomocysteinemia induces hepatic cholesterol biosynthesis and lipid accumulation via activation of transcription factors. Am. J. Physiology Endocrinol. Metabolism 288 (5), E1002–E1010. 10.1152/ajpendo.00518.2004 15644462

[B49] Yao Y. S. Li T.Di Zeng Z. H. (2020). Mechanisms underlying direct actions of hyperlipidemia on myocardium: an updated review. Lipids Health Dis. 19 (1), 23–26. 10.1186/s12944-019-1171-8 32035485 PMC7007679

[B50] Zanwar A. A. Hegde M. V. Rojatkar S. R. Sonawane K. B. Rajamohanan P. R. Bodhankar S. L. (2014). Isolation, characterization and antihyperlipidemic activity of secoisolariciresinol diglucoside in poloxamer-407-induced experimental hyperlipidemia. Pharm. Biol. 52 (9), 1094–1103. 10.3109/13880209.2013.877492 24649910

[B51] Zhang K. Kaufman R. J. (2008). From endoplasmic-reticulum stress to the inflammatory response. Nature 454 (7203), 455–462. 10.1038/nature07203 18650916 PMC2727659

[B52] Zhou J. Austin R. C. (2009). Contributions of hyperhomocysteinemia to atherosclerosis: causal relationship and potential mechanisms. BioFactors 35 (2), 120–129. 10.1002/BIOF.17 19449439

[B53] Zipes D. P. Libby P. Bonow R. O. Mann D. L. Tomaselli G. F. (2019). Braunwald’s heart disease: a textbook of cardiovascular medicine, 2 Set. 82–128.

