# Supplementary material for: Linagliptin and secoisolariciresinol diglucoside attenuate hyperlipidemia and cardiac hypertrophy induced by a high-methionine diet in rats via suppression of hyperhomocysteinemia-induced endoplasmic reticulum stress
Source: Front Pharmacol. 2023 Nov 9;14:1275730. doi: 10.3389/fphar.2023.1275730 (PMC10665493; doi:10.3389/fphar.2023.1275730)
Supplement: Supplementary file 1 [file Table1.DOCX]

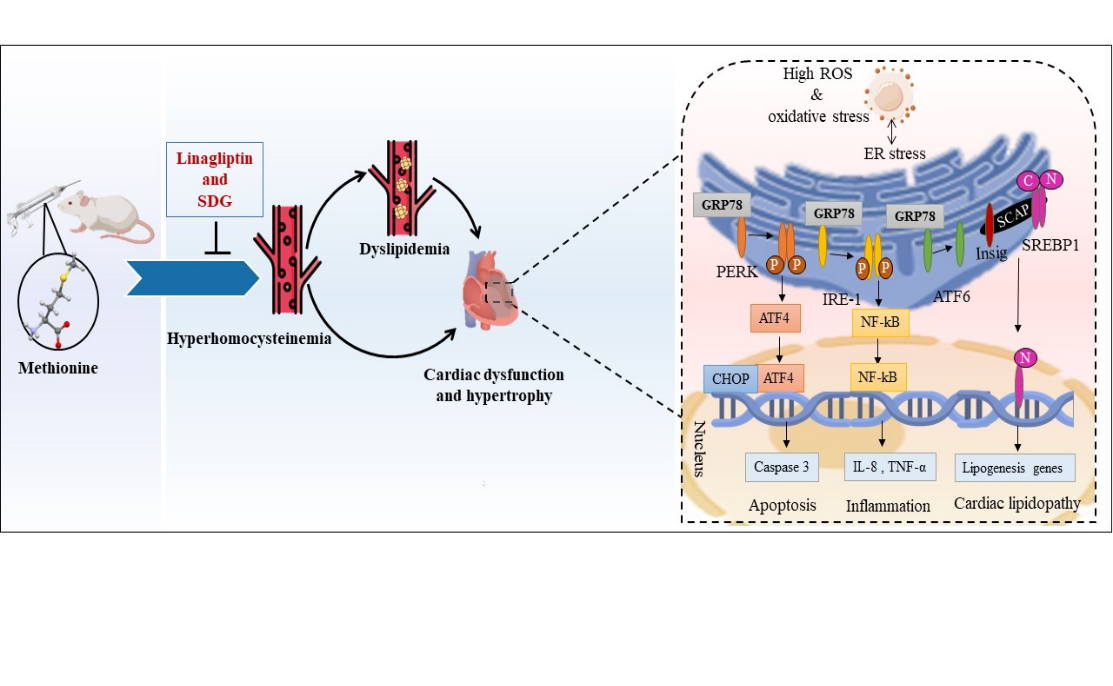


The possible protective effect of Linagliptin, SDG, and their combination against hyperlipidemia and cardiac hypertrophy induced by hyperhomocysteinemia in rats via reducing endoplasmic reticulum (ER) stress.

Abbreviations:

SDG: Secoisolariciresinol Diglucoside; Glucose- regulated protein 78 (GRP78); SREBPs: Sterol regulatory element-binding proteins; IRE1: Inositol-requiring enzyme 1 ; PERK: Protein kinase RNA- like Endoplasmic reticulum kinase; ATF 6: activating transcription factor 6; CHOP: C/EBP homologous protein; NF-kB:nuclear factor kappa-b; TNF-α: tumor necrosis factor alpha; IL 8: interleukin-8.
